# Health of Pug dogs in the UK: disorder predispositions and protections

**DOI:** 10.1186/s40575-022-00117-6

**Published:** 2022-05-18

**Authors:** Dan G. O’Neill, Jaya Sahota, Dave C. Brodbelt, David B. Church, Rowena M.A. Packer, Camilla Pegram

**Affiliations:** 1 Pathobiology and Population Sciences, The Royal Veterinary College, Hawkshead Lane, North Mymms, Hatfield, Herts AL9 7TA UK; 2 Clinical Science and Services, The Royal Veterinary College, Hawkshead Lane, North Mymms, Hatfield, Herts AL9 7TA UK

**Keywords:** VetCompass, Electronic patient record, EPR, Breed, Dog, Epidemiology, Primary-care, Veterinary, Pedigree, Purebred, Pug

## Abstract

**Background:**

Pugs are a brachycephalic dog breed that has become phenomenally popular over recent decades. However, there is growing concern about serious health and welfare issues in the breed. To augment the evidence-base on the comparative health of Pugs, this study aimed to compare the odds of common disorders between Pugs and all remaining dogs under primary veterinary care in the UK during 2016.

A cross-sectional study design of VetCompass clinical records was used to estimate the one-year (2016) period prevalence for the disorders most commonly diagnosed in Pugs and non-Pugs. Risk factor analysis applied multivariable logistic regression modelling methods to compare the odds of 40 common disorders between Pugs and non-Pugs.

**Results:**

From a study population of 905,544 dogs, the analysis included random samples of 4308 Pugs and 21,835 non-Pugs. Pugs were younger (2.36 years, range 0.07–16.24 vs 4.44 years, range 0.01–20.46, *p* <  0.001) and lighter (8.95 kg, range 5.00–13.60 vs. 14.07 kg, range 1.41–85.00, *p* <  0.001) than non-Pugs. Pugs had 1.86 (95% confidence interval [CI] 1.72 to 2.01) times the adjusted odds of diagnosis with ≥1 disorder than non-Pugs. Pugs had significantly increased adjusted odds for 23/40 (57.5%) common disorders. These included: brachycephalic obstructive airway syndrome (odds ratio [OR] 53.92; 95% CI 36.22 to 80.28), stenotic nares (OR 51.25; 95% CI 24.93 to 105.37) and corneal ulceration (OR 13.01; 95% CI 10.50 to 16.11). Conversely, Pugs had significantly reduced adjusted odds of 7/40 (17.5%) common disorders compared to non-Pugs. These included: heart murmur (OR 0.23; 95% 0.13 to 0.14), lipoma (OR 0.24; 95% CI 0.11 to 0.55) and aggression (OR 0.31; 95% CI 0.21 to 0.47).

**Conclusions:**

The current study highlights that predispositions outnumber protections between Pugs and non-Pugs for common disorders, suggesting some critical health welfare challenges to overcome for Pugs. Highly differing heath profiles between Pugs and other dogs in the UK suggest that the Pug has diverged substantially from mainstream dog breeds and can no longer be considered as a typical dog from a health perspective.

## Plain English summary

Pugs have become phenomenally popular in the UK over recent decades. The breed has a flat-faced look (brachycephalic) that many humans find highly attractive and ‘cute’ but this flat face is also linked to several serious health problems. Consequently, there is growing concern about the welfare issues associated with the popularity and health issues of Pugs. To get a better overall perspective on the health of Pugs, this study aimed to compare the risks of common disorders between Pugs and all remaining dogs.

The study collected clinical information from first opinion veterinary practices in the UK that were participating in VetCompass. Dogs were grouped as either Pugs or non-Pugs. Information was gathered from the clinical records on all disorders diagnosed in each group during 2016 and a list of the most common disorders in each group was generated.

From 905,544 dogs in the overall study, there were 16,218 (1.79%) Pugs and 889,326 (98.21%) non-Pugs. Pugs (2.36 years, interquartile range [IQR] 1.16–4.53) were generally younger than non-Pugs (4.44 years, IQR 1.90–8.12). Pugs (8.95 kg, IQR 7.80–10.17) were also generally lower in bodyweight than non-Pugs (14.07 kg, IQR 8.15–25.20). From a combined list of 40 common disorders among both groups of dogs, Pugs had increased risk for 23 (57.5%) but had reduced risk for 7 (17.5%) disorders compared to non-Pugs. The disorders with the highest relative risk in Pugs included brachycephalic obstructive airway syndrome (BOAS) (× 53.92 risk), narrowed nostrils (× 51.25), eye ulceration (× 13.01), and skin fold infection (× 10.98). Disorders with the lowest relative risk in Pugs included heart murmur (× 0.23), fatty lump (× 0.24), aggression (× 0.31), and wounds (× 0.53).

The study provides a broad evidence base on the positive and negative aspects of the health of Pugs. Disease predispositions were more common than disease protections, confirming the hypothesis that there are many critical health-related welfare challenges to overcome for Pugs. The widely differing health profiles between Pugs and other dogs in the UK suggest that the Pug has now diverged to such an extent from mainstream dog breeds that it can no longer be considered as a typical dog from a health perspective.

## Background

Artificial selection has led to the domestic dog, *Canis lupis familiaris*, becoming the most phenotypically distinct mammalian species on the planet [1, 2]. However, distinctly differing health profiles have emerged across the spectrum of modern dog breeds that are closely aligned with common conformational features selected by humans such as brachycephaly [3–5]. Breeds such as Pugs, French Bulldogs and English Bulldogs with a brachycephalic (flat-faced) conformation have become phenomenally popular in the UK over recent decades [6–9]. However, there is growing concern from charity and welfare groups about serious health and welfare issues associated with these popular brachycephalic breeds [10, 11] based on an expanding worldwide evidence base [5, 12]. Reflecting these concerns, the current advice for the general public from the UK Brachycephalic Working Group, which comprises members from many of the major UK dog welfare stakeholders, is to ‘Stop and think before buying a flat-faced dog’ [13].

Pugs are thought to represent an ancient dog morphology, with ‘short-mouthed’ dogs that looked similar to the modern Pug documented by Confucius as early as 551 BC [14, 15]. The name of the breed derives from *pugnus,* the Latin for *fist,* because the side head profile resembles the shape of a closed fist [14, 15]. The popularity of Pugs has risen sharply over the past two decades, with the UK Kennel Club annual registration data showing a rise from 2116 registrations in 2005 to 6033 registrations in 2020, although their popularity seems to have waned recently [9].

The Pug’s small bodysize, combined with a flat face, large head, bulging eyes and wrinkled forehead, may arouse positive emotions by conforming to the ‘baby schema’ facial configuration which triggers innate nurturing responses in humans [16]. Lorenz noted that breeds of dog, such as the Pug, have retained infant-like features into adulthood [16], and empirical studies have since demonstrated that these features increase attractiveness, particularly for women [17]. Recent research found that owners of Pugs exhibited stronger higher emotional closeness with their dog than owners of other brachycephalic breeds, with these emotional bonds particularly high in female owners [18].

However, these same facial features that humans find so appealing have also been widely associated with several conformationally-driven disorders including brachycephalic obstructive airway syndrome (BOAS) [19], corneal ulceration [20], dystocia [21] and upper respiratory tract disorders [22]. In line with a breed standard that states that the Pug must ‘never to appear low on legs, nor lean and leggy’ [23], obesity has been reported as the most common disorder of Pugs and also that Pugs are the dog breed with the highest predisposition for obesity in the UK [24, 25]. Predispositions to 41 disorders have been reported worldwide in Pugs [4].

The Kennel Club’s ‘Breed Health and Conservation Plan’ project aims to redress some of these issues in the pedigree subset of UK Pugs by providing a collaborative basis to create evidence-based strategies to tackle the most important health priorities [26]. The 2020 Breed Health and Conservation Plan for Pugs has identified BOAS, epilepsy, eye conditions, obesity, Pug dog encephalitis, skin conditions and spinal problems as priority health issues for the breed [27].

However, despite a growing evidence base, there are still information gaps about the health of Pugs relative to the general population of dogs. Many of the previous studies focused on single disorders rather than across a range of disorders [20] or were based on biased subsets of dogs such as referral populations or the pedigree registered subsets of dogs [28]. Even those studies that were based on the wider population and across the full range of disorders tended to offer descriptive rather than comparative analyses [24]. In addition, many of the earlier studies applied univariable analytic methods that failed to account for important confounding effects from differences such as age and bodyweight between Pug and non-Pug populations [5, 24]. Much of the previous research in canine health has focused on identifying disorder predispositions (i.e., increased susceptibility to certain disorders) [4]. However, it is becoming increasingly apparent that greater exploration of disorder protections (i.e., resistance to developing certain disorders) is needed because understanding why breeds do not develop certain disorders may be as, or even more, important as understanding why they do develop these disorders [29, 30]. With this background in mind, the current study aimed to explore anonymised veterinary clinical data from the VetCompass Programme [31] to compare the odds of common disorders between Pugs and all remaining dogs under primary veterinary care in the UK during 2016 after accounting for major confounding variables. The objectives were to identify a list of common disorders with predisposition and protection in the Pug. These results could assist breeders, veterinary practitioners and owners with a more reliable evidence base on the health of the wider population of Pugs in the UK to predict, prevent and manage key health and welfare opportunities for the breed. Based on the prior body of evidence suggesting high levels of predispositions in Pugs, the current study hypothesized that the count of disorder predispositions would outnumber the count of disorder protections in Pugs.

## Methods

The data extraction, collation and analytic methods used in this study are deliberately similar to other VetCompass studies in order to promote comparability across the study outputs [29, 30, 32]. The study population included all available dogs under primary veterinary care at 886 clinics participating in the VetCompass Programme during 2016. Dogs under veterinary care had either a) ≥1 electronic patient record (EPR) during 2016 or b) ≥1 EPR during both 2015 and 2017 [31]. The data fields included a unique animal identifier along with species, breed, date of birth, sex, neuter status, insurance status and bodyweight, and also clinical information from free-form text clinical notes and treatment with relevant dates [33].

A cross-sectional study design was used to estimate and compare the one-year (2016) period prevalence of the most commonly diagnosed disorders between random samples of Pugs and all other dogs. Power calculations estimated that 3092 Pugs and 18,549 non-Pugs were required to detect an odds ratio of ≥1.5 for a disorder occurring in 1.5% of the non-Pugs, with 80% power and 95% confidence and assuming a 6:1 ratio of non-Pugs to Pugs in the study population [24, 34]. Ethics approval was obtained from the RVC Ethics and Welfare Committee (reference number SR2018–1652).

Breed information entered by the participating practices was cleaned and mapped to a VetCompass breed list derived from the VeNom Coding breed list [35]. Dogs recorded as Pug were categorised as Pug and dogs recorded with any other breed or crossbred term were categorised as non-Pug. Neuter and insurance status was defined at the final available EPR. Adult bodyweight described the mean of all bodyweight (kg) values recorded for each dog after reaching 18 months old. Mean adult bodyweight was reported by sex for all breeds with adult bodyweight available for at least 100 dogs. Bodyweight was further categorized as “at or above the breed/sex mean”, “below the breed/sex mean” and “no recorded bodyweight”. Age (years) at the final study date (December 31, 2016) was categorised: < 1.0, 1.0 to < 2.0, 2.0 to < 4.0, 4.0 to < 6.0, 6.0 to < 8.0 and ≥ 8.0. The veterinary group attended by the study animals were distributed throughout the UK and were categorised as 1–5 for anonymity.

The clinical records of randomly selected subsets of Pugs and non-Pugs were reviewed to extract the most definitive diagnoses recorded for all disorders with evidence of existence during 2016 as previously described [33]. Randomisation was applied within the VetCompass online database (https://www.vetcompass.org) using the built-in Microsoft function RAND (Transact-SQL). Elective (e.g., neutering) or prophylactic (e.g., vaccination) clinical events were not included. Disorders described within the clinical notes using presenting sign terms (e.g., ‘vomiting’ or ‘vomiting and diarrhoea’), but without a formally recorded clinical diagnostic term, were included using the first sign listed (e.g., vomiting). The extracted diagnosis terms were mapped to a dual hierarchy of diagnostic precision for analysis: specific-level precision (maximal diagnostic precision recorded within the clinical notes) and grouped-level precision (general level of diagnostic precision) as previously described [33].

Following data checking for internal validity and cleaning in Excel (Microsoft Office Excel 2013, Microsoft Corp.), analyses were conducted using SPSS version 24.0 (IBM Corp). The sex-neuter status, age, adult bodyweight and insurance status for Pugs and non-Pugs under veterinary care during 2016 were described. One-year period prevalence described the probability of diagnosis at least once during 2016 within Pugs and non-Pugs [33]. All disorders among the 30 most common disorders for Pugs and for non-Pugs were combined into an overall list of common disorders that included 40 specific-level disorders and 32 grouped-level disorders. Non-normally distributed continuous variables were summarised using median, interquartile range (IQR) and range. Univariable statistical comparisons used Mann-Whitney U test and chi-square test as appropriate [36]. Multivariable binary logistic regression modelling was used to report the adjusted odd ratios (aOR) comparing Pugs with non-Pugs for each disorder in the combined lists of common disorders. A separate model was created for each individual specific-level and grouped disorder. Information theory was applied to generate a list of confounding variables that were consistently included alongside the breed variable in each model [37, 38]. Breed was an a priori factor of interest and each model additionally included age (years), sex-neuter status, at/above or below mean bodyweight, insurance status and veterinary group. Statistical significance was set at the 5% level. At a summary level of disorder diagnosis, dogs were binary-classified with either no disorder or ≥ 1 disorder during 2016. The multivariable modelling approach above was also used to report the odds of diagnosis with ≥1 disorder during 2016 in Pugs compared with non-Pugs.

## Results

The study population of 905,544 dogs under veterinary care during 2016 in the UK included 16,218 (1.79%) Pugs and 889,326 (98.21%) non-Pugs. Random samples of 4308/16,218 (26.56%) Pugs and 21,835/889,326 (2.46%) non-Pugs were included in the analysis. Data completeness were breed 99.7%, age 98.8%, sex-neuter status 99.7%, insurance status 100.0% and bodyweight 65.4%.

Descriptive results were reported on 4308 Pugs and 21,835 non-Pugs (Table 1). The median age of Pugs (2.36 years, IQR 1.16–4.53, range 0.07–16.24) was substantially and significantly younger than for non-Pugs (4.44 years, IQR 1.90–8.12, range 0.01–20.46) (*p* <  0.001). The median bodyweight of Pugs (8.95 kg, IQR 7.80–10.17, range 5.00–13.60) was substantially and significantly lighter than for non-Pugs (14.07 kg, IQR 8.15–25.20, range 1.41–85.00) (*p* <  0.001).

**Table 1:** Descriptive statistics for demographic characteristics in Pugs (*n* = 4308) and non-Pugs (*n* = 21,835) under primary veterinary care in the UK. The *P*-value represents comparison of demographic variables between Pugs and non-Pugs

Variable	Category	Pug count (%)	Non-Pug count (%)	*P*-value
Age (years)	< 1.0	855 (20.1)	2413 (11.2)	< 0.001
	1.0 to < 2.0	1031 (24.2)	3186 (14.8)	
	2.0 to < 4.0	1109 (26.1)	4340 (20.1)	
	4.0 to < 6.0	598 (14.1)	3383 (15.7)	
	6.0 to < 8.0	350 (8.2)	2748 (12.7)	
	≥ 8.0	311 (7.3)	5521 (25.6)	
Sex-neuter status	Male entire	1583 (36.9)	6318 (29.0)	< 0.001
	Male neutered	702 (16.4)	5154 (23.7)	
	Female entire	1433 (33.4)	5517 (25.3)	
	Female neutered	575 (13.4)	4780 (22.0)	
At/above or below mean bodyweight for breed and sex	At or above	1163 (27.0)	6402 (29.3)	< 0.001
	Below	1300 (30.2)	8248 (37.8)	
	Not recorded	1845 (42.8)	7185 (32.9)	
Insurance status	Insured	546 (12.7)	2912 (13.3)	0.241
	Not insured	3762 (87.3)	18,923 (86.7)	
Vet Group	1	2000 (46.4)	9851 (45.1)	< 0.001
	2	1518 (35.2)	7174 (32.9)	
	3	618 (14.3)	3742 (17.1)	
	4	138 (3.2)	994 (4.6)	
	5	34 (0.8)	74 (0.3)	

Of the Pugs, 3164/4308 (73.4%) were diagnosed with ≥1 disorder compared with 14,408/21,835 (66.0%) of the non-Pugs. The remaining dogs not diagnosed with at least one disorder either received prophylactic care or did not visit the veterinary practice during 2016. Multivariable modelling identified that Pugs had 1.86 (95% confidence interval [CI] 1.72 to 2.01) times the adjusted odds of diagnosis with ≥1 disorder than non-Pugs (*p* <  0.001).

At a specific-level of diagnostic precision, after accounting for confounding using multivariable methods, Pugs had significantly increased adjusted odds of 23/40 (57.5%) specific-level disorders compared to non-Pugs. These included: BOAS (odds ratio [OR] 53.92; 95% CI 36.22 to 80.28; *p* <  0.001), stenotic nares (OR 51.25; 95% CI 24.93 to 105.37; *p* <  0.001), corneal ulceration (OR 13.01; 95% CI 10.50 to 16.11; *p* <  0.001), skin fold dermatitis (OR 10.98; 95% CI 7.64 to 15.76; *p* <  0.001) and aural discharge (OR 9.61; 95% CI 6.36 to 14.53; *p* <  0.001). Conversely, Pugs had significantly reduced adjusted odds of 7/40 (17.5%) specific-level disorders compared to non-Pugs. These included: heart murmur (OR 0.23; 95% 0.13 to 0.14; *p* <  0.001), lipoma (OR 0.24; 95% CI 0.11 to 0.55; *p* = 0.001), aggression (OR 0.31; 95% CI 0.21 to 0.47; *p* <  0.001), wounds (OR 0.53; 95% CI 0.38 to 0.72; *p* <  0.001) and foreign body (OR 0.62; 95% CI 0.44 to 0.87; *p* = 0.006) (Table 2).

**Table 2:** Multivariable logistic regression odds ratios with corresponding 95% CI (confidence interval) for the combined list from the 30 most common disorders in Pugs and the 30 most common disorders in non-Pugs at a *specific-level of diagnostic precision* recorded in dogs under primary veterinary care at UK practices participating in the VetCompass™ Programme from January 1st 2016 to December 31st, 2016. The results are adjusted for the effects of age, sex-neuter status, at/above or below mean bodyweight, insurance status and vet group. Specific-level precision describes the original extracted terms at the maximal diagnostic precision recorded within the clinical notes

Specific-level disorder	Pug Count (%)	Non-Pug Count (%)	Odds ratio	95% CI^a^	*P*-value
Brachycephalic obstructive airway syndrome	285 (6.6)	29 (0.1)	53.92	36.22 to 80.28	**< 0.001**
Stenotic nares	116 (2.7)	8 (0.0)	51.25	24.93 to 105.37	**< 0.001**
Corneal ulceration	294 (6.8)	153 (0.7)	13.01	10.50 to 16.11	**< 0.001**
Skin fold dermatitis	101 (2.3)	51 (0.2)	10.98	7.64 to 15.76	**< 0.001**
Aural discharge	77 (1.8)	36 (0.2)	9.61	6.36 to 14.53	**< 0.001**
Allergic skin disorder	69 (1.6)	64 (0.3)	5.88	4.10 to 8.42	**< 0.001**
Demodicosis	72 (1.7)	45 (0.2)	5.61	3.82 to 8.26	**< 0.001**
Retained deciduous tooth	235 (5.5)	210 (1.0)	4.31	3.54 to 5.24	**< 0.001**
Obesity	751 (17.4)	1515 (6.9)	3.89	3.51 to 4.32	**< 0.001**
Umbilical hernia	200 (4.6)	189 (0.9)	3.72	3.02 to 4.59	**< 0.001**
Ocular discharge	83 (1.9)	157 (0.7)	2.55	1.93 to 3.38	**< 0.001**
Overgrown nails	507 (11.8)	1198 (5.5)	2.55	2.27 to 2.86	**< 0.001**
Cryptorchidism	89 (2.1)	124 (0.6)	2.52	1.89 to 3.35	**< 0.001**
Patellar luxation	93 (2.2)	219 (1.0)	2.26	1.75 to 2.92	**< 0.001**
Anal sac impaction	378 (8.8)	1040 (4.8)	2.23	1.96 to 2.54	**< 0.001**
Alopecia	75 (1.7)	178 (0.8)	2.19	1.65 to 2.90	**< 0.001**
Otitis externa	556 (12.9)	1579 (7.2)	2.04	1.83 to 2.28	**< 0.001**
Coughing	69 (1.6)	210 (1.0)	1.94	1.45 to 2.59	**< 0.001**
Post-operative wound	98 (2.3)	263 (1.2)	1.82	1.43 to 2.33	**< 0.001**
Pyoderma	87 (2.0)	304 (1.4)	1.57	1.22 to 2.02	**< 0.001**
Vomiting	194 (4.5)	666 (3.1)	1.41	1.19 to 1.67	**< 0.001**
Conjunctivitis	127 (2.9)	490 (2.2)	1.31	1.07 to 1.61	**0.010**
Atopic dermatitis	51 (1.2)	249 (1.1)	1.26	0.92 to 1.72	0.146
Diarrhoea	215 (5.0)	839 (3.8)	1.19	1.02 to 1.40	**0.029**
Pododermatitis	56 (1.3)	294 (1.3)	1.11	0.83 to 1.49	0.494
Gastroenteritis	63 (1.5)	288 (1.3)	1.07	0.81 to 1.42	0.643
Kennel cough	47 (1.1)	214 (1.0)	1.05	0.76 to 1.45	0.793
Pruritus	67 (1.6)	359 (1.6)	0.98	0.75 to 1.29	0.903
Skin mass	51 (1.2)	459 (2.1)	0.94	0.69 to 1.26	0.662
Periodontal disease	315 (7.3)	2758 (12.6)	0.91	0.80 to 1.04	0.156
Flea infestation	85 (2.0)	450 (2.1)	0.89	0.70 to 1.13	0.340
Skin cyst	23 (0.5)	230 (1.1)	0.74	0.47 to 1.14	0.170
Osteoarthritis	28 (0.6)	519 (2.4)	0.71	0.48 to 1.06	0.091
Lameness	68 (1.6)	578 (2.6)	0.66	0.51 to 0.85	**0.002**
Claw injury	36 (0.8)	305 (1.4)	0.64	0.45 to 0.92	**0.016**
Foreign body	40 (0.9)	277 (1.3)	0.62	0.44 to 0.87	**0.006**
Wound	44 (1.0)	393 (1.8)	0.53	0.38 to 0.72	**< 0.001**
Aggression	27 (0.6)	489 (2.2)	0.31	0.21 to 0.47	**< 0.001**
Lipoma	6 (0.1)	320 (1.5)	0.24	0.11 to 0.55	**0.001**
Heart murmur	12 (0.3)	473 (2.2)	0.23	0.13 to 0.41	**< 0.001**

^a^*CI* confidence interval

At a grouped-level of diagnostic precision, after accounting for confounding using multivariable methods, Pugs had significantly increased adjusted odds of 23/32 (71.9%) grouped-level disorders compared to non-Pugs. These included: lower respiratory tract disorder (OR 7.50; 95% CI 5.81 to 9.68; *p* <  0.001), oral cavity disorder (OR 6.28; 95% CI 4.50 to 8.76; *p* <  0.001), upper respiratory tract disorder (OR 5.96; 95% CI 5.31 to 6.69; *p* <  0.001), abdominal disease (OR 5.48; 95% CI 3.84 to 7.82; *p* <  0.001) and brain disorder (OR 3.95; 95% CI 3.17 to 4.91; *p* <  0.001). Conversely, Pugs had significantly reduced odds of 2/32 (6.3%) grouped-level disorders compared to non-Pugs. These were: endocrine system disorder (OR 0.39; 95% CI 0.17 to 0.88; *p* = 0.024) and behaviour disorder (OR 0.50; 95% CI 0.41 to 0.61; *p* <  0.001) (Table 3).

**Table 3:** Multivariable logistic regression odds ratios with corresponding 95% CI (confidence interval) for the combined list from the 30 most common disorders in Pugs and the 30 most common disorders in non-Pugs at a *grouped-level of diagnostic precision* recorded in dogs under primary veterinary care at UK practices participating in the VetCompass™ Programme from January 1st 2016 to December 31st, 2016. The results are adjusted for the effects of age, sex-neuter status, at/above or below mean bodyweight, insurance status and vet group. Grouped-level precision describes the original extracted terms mapped to a general level of diagnostic precision

Grouped-level disorder	Pug Count (%)	Non-Pug Count (%)	Odds ratio	95% CI^a^	*P*-value
Lower respiratory tract disorder	143 (3.3)	143 (0.7)	7.50	5.81 to 9.68	**< 0.001**
Oral cavity disorder	77 (1.8)	90 (0.4)	6.28	4.50 to 8.76	**< 0.001**
Upper respiratory tract disorder	711 (16.5)	746 (3.4)	5.96	5.31 to 6.69	**< 0.001**
Abdominal disease	65 (1.5)	81 (0.4)	5.48	3.84 to 7.82	**< 0.001**
Brain disorder	143 (3.3)	306 (1.4)	3.95	3.17 to 4.91	**< 0.001**
Hernia	209 (4.9)	236 (1.1)	3.24	2.66 to 3.94	**< 0.001**
Ophthalmological disorder	679 (15.8)	1506 (6.9)	3.19	2.87 to 3.54	**< 0.001**
Collapsed	54 (1.3)	159 (0.7)	3.15	2.26 to 4.40	**< 0.001**
Urinary system disorder	104 (2.4)	257 (1.2)	3.03	2.37 to 3.88	**< 0.001**
Female reproductive system disorder	161 (3.7)	314 (1.4)	2.53	2.06 to 3.11	**< 0.001**
Congenital disorder	42 (1.0)	60 (0.3)	2.48	1.64 to 3.73	**< 0.001**
Appetite disorder	61 (1.4)	156 (0.7)	2.46	1.80 to 3.38	**< 0.001**
Male reproductive system disorder	113 (2.6)	200 (0.9)	2.41	1.88 to 3.08	**< 0.001**
Ear disorder	651 (15.1)	1775 (8.1)	2.17	1.96 to 2.41	**< 0.001**
Claw/nail disorder	542 (12.6)	1538 (7.0)	2.12	1.90 to 2.36	**< 0.001**
Anal sac disorder	385 (8.9)	1217 (5.6)	1.97	1.74 to 2.23	**< 0.001**
Spinal cord disorder	46 (1.1)	210 (1.0)	1.93	1.38 to 2.71	**< 0.001**
Incontinence	30 (0.7)	187 (0.9)	1.90	1.26 to 2.85	**0.002**
Complication associated with clinical care	149 (3.5)	405 (1.8)	1.75	1.44 to 2.13	**< 0.001**
Lethargy	77 (1.8)	270 (1.2)	1.65	1.26 to 2.16	**< 0.001**
Skin disorder	721 (16.7)	2755 (12.6)	1.51	1.38 to 1.66	**< 0.001**
Dental disorder	577 (13.4)	3082 (14.1)	1.42	1.28 to 1.57	**< 0.001**
Adverse reaction to drug	42 (1.0)	162 (0.7)	1.16	0.82 to 1.65	0.404
Mass	147 (3.4)	1130 (5.2)	1.13	0.94 to 1.36	0.204
Parasite infestation	217 (5.0)	833 (3.8)	1.13	0.97 to 1.33	0.120
Enteropathy	529 (12.3)	2291 (10.5)	1.12	1.00 to 1.24	**0.037**
Neoplasia	132 (3.1)	1125 (5.1)	1.07	0.88 to 1.30	0.480
Heart disease	67 (1.6)	643 (2.9)	0.96	0.74 to 1.25	0.759
Musculoskeletal disorder	263 (6.1)	1898 (8.7)	0.96	0.83 to 1.10	0.528
Traumatic injury	145 (3.4)	809 (3.7)	0.84	0.69 to 1.01	0.058
Behaviour disorder	108 (2.5)	1122 (5.1)	0.50	0.41 to 0.61	**< 0.001**
Endocrine system disorder	6 (0.1)	191 (0.9)	0.39	0.17 to 0.88	**0.024**

^a^*CI* confidence interval

## Discussion

Based on a background of high popularity combined with high concern for the health and welfare of Pugs, this study explored UK primary-care veterinary data to identify predispositions and protections among common disorders in Pugs and non-Pugs. Pugs were generally younger and lighter than non-Pugs in the UK and hence multivariable methods were used to adjust for demographic confounding effects [5]. In line with the study hypothesis, the study identified substantially more predispositions than protections for Pugs, supporting prior reports of poor health status for Pugs overall. However, the paper also highlights some important disorder protections that may help to explain the enduring popularity of the breed [9].

Predisposition to disorders associated with selection towards extremes of conformation in dog breeds have been reported since Charles Darwin theorised in 1868 that muscular defects in Scottish Deerhounds were related to their giant size [39]. Since then, a growing and large body of evidence has accumulated on breed predispositions related to conformation in dogs [3, 4]. Conversely, the extent of the health differences between a breed and all remaining dogs could be taken as a measure of divergence of that breed from the mainstream of dogs [32]. With this perspective, the current study suggests that Pugs have diverged substantially from other dogs because Pugs showed differing odds (either predisposition or protection) for 30/40 (75%) of common disorders. Given that 23/30 (76.7%) of these differences were predispositions rather than protections, this further suggests that divergence from mainstream dog characteristics in Pugs has selected towards greater negative than positive effects for the breed. The results for the French Bulldog, another breed with extreme brachycephaly, were similar to those for the Pug, showing French Bulldogs differed to other dogs in 31/43 (72.1%) of common disorders, showing predispositions in 20/31 (64.5%) and protections in 11/31 (35.5%) [32]. Studies with similar design to the current study have previously reported that Labrador Retrievers differed to other dogs in 19/35 (54.3%) of common disorders, showing predispositions in 12/19 (63.2%) and protections in 7/19 (36.8%) [30]. Staffordshire Bull Terriers differed to other dogs in 9/36 (25.0%) of common disorders, showing predispositions in 4/9 (44.4%) and protections in 5/9 (55.6%) [29]. The current findings support the concerns of large welfare collaborations, such as the UK Brachycephalic Working Group [13], that many Pugs suffer from seriously compromised health and welfare related to their breed. Although efforts are underway to redress some of these issues by health programmes such as the Kennel Club’s ‘Breed health and Conservation Plan’ project [26] and the Pug Breed Council’s ‘Pug 5 Star health Scheme’, the advice in the meantime from the Brachycephalic Working Group is for prospective owners to ‘Stop and think before buying a flat-faced dog’ [13]. Future work that aims for deeper understanding of the overall conformation-related welfare burden could additionally aim to explore the effects from severity and duration of these predisposed disorders in Pugs [40].

Deeper understanding of the innate health risks associated with extreme brachycephaly can be gained by comparison of ultra-predispositions between breeds with extreme brachycephaly such as Pugs and French Bulldogs. Ultra-predispositions have been defined as disorders with odds over 4 times higher in a breed compared with all other dogs not of that breed [32]. Exploration of ultra-predispositions among common disorders offer insights into the key health welfare issues associated that unique breed. In the current study, Pugs are reported with 8 ultra-predispositions among 30 common disorders (26.7%). French Bulldogs have previously been reported with a remarkably similar proportion (25.6%), showing 11 ultra-predispositions among 43 common disorders [32]. Even more remarkable is the finding that 7 of the 8 ultra-predispositions in Pugs are shared as ultra-predispositions in French Bulldogs: BOAS, stenotic nares, corneal ulceration, skin fold dermatitis, aural discharge, allergic skin disorder and demodicosis. These similarities in ultra-predispositions between two breeds that have similarly been selected by humans for extreme brachycephaly add further weight to the growing evidence base on the health welfare harms associated with extreme brachycephaly [13, 23, 41].

Obesity was the most common disorder in Pugs, affecting 17.4% of the Pugs compared with just 6.9% of the non-Pugs. This high prevalence concurs with the findings of an earlier study of first opinion clinical records that similarly highlighted obesity as the most common disorder in Pugs in the UK [24]. Pugs were also strongly predisposed to obesity, showing 3.89 times the adjusted odds compared with non-Pugs in the current study. Overweight and obesity bring severe welfare consequences for affected dogs, including shortened life span [42, 43], reduced quality of life [44, 45] and increased risk for osteoarthritis, diabetes mellitus and neoplasia [46]. Obesity presents additional challenges specifically for Pugs by compounding their intrinsically high predisposition for BOAS [47, 48]. As a consequence, it may be prudent to immediately revise the Pug Kennel Club breed standard by replacing the current wording that the breed should ‘never to appear low on legs, nor lean and leggy’ with alternative wording that prioritises health over human appeal by promoting leanness in the breed [23]. It is worth noting that absolute bodyweight in kilograms should not be conflated with body condition score [25]. The current study reports that Pugs are highly predisposed to obesity despite having significantly lower median absolute adult bodyweight compared to non-Pugs (8.95 kg, vs 14.07 kg respectively).

 Reduced risk of aggression (OR 0.31) featured highly among the 7/40 disorder protections reported here in Pugs. French bulldogs, another extreme brachycephalic breed with huge popularity, were similarly reported recently with protection to aggression (OR 0.64) [32]. The Kennel Club breed standard describes Pugs as “even tempered, happy and lively disposition” [23]. In both owner-reported and practical tests of dog behaviour, brachycephalic dogs are reported to be more affectionate, cooperative and interactive with unfamiliar humans than longer-muzzled dogs [49, 50]. Such positive behavioural traits may drive owner attraction and engender loyalty towards the breed, with recent qualitative research finding that owners of brachycephalic breeds (including the Pug) recommended their breed based on their loving and affectionate natures, being comical or ‘clown like’, playful and easily trained [51]. The commonality in ‘protection’ against aggression across brachycephalic breeds may reflect shared genetic factors underpinning dog behaviour, but may also reflect shared perceptual differences of their owners, given that this ownership group are known to have divergent assessments of dog health and welfare compared with veterinary professionals [18, 51]. Although Pugs are often considered by owners as the ideal companion dog, particularly for households with children [51, 52], and were rated a ‘very low’ aggressive breed by veterinary surgeons in New Zealand [53], there is limited evidence outside of human perceptions that Pugs are less aggressive than other dogs breeds. Furthermore, there is limited robust evidence that breed per se is a risk or protective factor for dog bites [54]. Consequently, caution should be exercised before promoting Pugs as superior family pets based on perceived lower aggression [55]).

Pugs are considered as a breed with extreme brachycephaly, with very low craniofacial ratios (CFR) that are evident phenotypically as a very flat face in the living dogs (mean CFR = 0.08 in two separate UK populations) [19, 48]. The extremely flattened faces and heads of Pugs have been associated with respiratory disorders, including BOAS [19, 48], as well as upper respiratory tract disorders in general [22, 56]. As a brachycephalic breed, although it was unsurprising to find a higher prevalence and odds ratio for BOAS in the current study for Pugs, the scale of these differences was notable. Pugs represented 6.6% of dogs recorded with BOAS in a single year compared with just 0.1% of non-Pugs, and Pugs were 53.92 times more likely to have BOAS compared with non-Pugs. Stenotic nares, a common anatomical component of the BOAS syndrome [19, 57], was also highly predisposed in Pugs (odds ratio 51.25), with 2.7% of Pugs affected compared with almost none of the non-Pugs. Indeed, BOAS and stenotic nares represented the two disorders with the highest predispositions in Pugs.

Despite worryingly high prevalence of BOAS, a condition with a high welfare impact [48], it is likely that the true prevalence of BOAS in Pugs is much higher than reported in the current study. There is strong evidence that the dyspnoea, snoring, air hunger and sleep apnoea that are typical clinical signs of BOAS are widely normalised within the breed (and also in other brachycephalic breeds) and therefore accepted as just being part of ‘what makes a Pug a Pug’ and adds to the experience of humans of owning a Pug [18, 51, 58]. Perhaps of even greater concern is that some of these pathological signs are celebrated as desirable characteristic of Pugs, including perceived ‘laziness’ [51]. Although ‘laziness’ may reflect exercise intolerance associated with BOAS [59], a perceived need for low levels of exercise is often promoted as a selling feature of the breed to prospective owners with a sedentary lifestyle, limited time to exercise their dog and limited space [51]. There are now growing efforts to move away from earlier anthropocentric approaches to the design of dog breeds that we now own and to move towards a new zeitgeist whereby the needs of the dog are always firmly prioritised over the desires of owners [26]. The stated view of the UK Brachycephalic Working Group that represents the mainstream of major dog welfare stakeholders in the UK is that ‘maximising good health, welfare and temperament overrides all other considerations for dogs’ [13]. Living up to this aspirational goal will require substantial human behavioural and legislative change to move away from seeing dogs as commodities that can be shaped to suit humans, and instead towards seeing dogs as sentient beings whose welfare we, as humans, are responsible for [60].

Corneal ulceration represented the third highest predisposition in Pugs in the current study, and, with an odds ratio of 13.01, should be considered as an ultra-predisposition (odds ratio greater than 4.0) in Pugs [32]. There were 6.8% of Pugs affected with corneal ulceration compared with just 0.7% of non-Pugs. In a previous study of corneal ulceration based on primary-care clinical records, the Pug was the breed with the highest prevalence with 5.4% of dogs affected [20]. Conformation risk factors that are common in Pugs have been reported for corneal ulceration, including brachycephalic craniofacial conformation, scleral exposure, and excessive nasal folds [48]. The UK Kennel Club has recognised and responded to the health consequences from these conformational issues by moving Pugs to category-3 Breedwatch to discourage features such as excessive nasal folds, excessively prominent eyes, incomplete blink and sore eyes due to damage or poor eyelid conformation [9].

It is noteworthy that the median age of Pugs (2.36 years) in the current study differed markedly from non-Pugs (4.44 years). There is substantial previous evidence that advancing age is a major risk factor for the majority of common disorders affecting dogs [25, 61]. A study of the 70 most common disorders among a sample of 22,333 dogs under primary veterinary care in the UK reported that only 13/70 (18.6%) disorder showed a median age of affected dogs that was below the median overall age of the study population (4.4 years) [33]. For this reason, earlier studies that have applied univariable statistical methods to compare the odds of disorders between groups of dogs with widely differing ages are likely to have been heavily confounded and therefore to have led to unsafe inference [5]. In an effort to mitigate these risk of confounding by age and other differing demography between Pugs and non-Pugs, the current study applies multivariable statistical modelling to account for these differences [62]. However, although this approach may have accounted for confounding effects on the current age cohort of Pugs, it is still possible that these methods may have underestimated the future risk of disorders for this cohort of Pugs as these dogs age. It is also worth considering the possible explanations for the relatively younger ages of the current Pug population compared with non-Pugs. This relative youth of Pugs may be partially explained by recent surge in popularity for Pugs and thus by a rapid influx of puppies entering the overall population [9, 24]. However, there is growing evidence on the life-limiting effects of many of the disorder predispositions that are reported in Pugs and therefore the overall low median ages of Pugs may also be contributed to by high levels of deaths at relatively young ages in Pugs [4, 5].

This study had several limitations consistent with VetCompass publications that have been previously reported and that are largely based on the nature of retrospective analysis of electronic patient record data [63, 64]. In addition, it is noteworthy that disorder profiles in Pugs in the current study were compared against a residual sample of dogs that included many other brachycephalic breeds. Given that 18.74% of dogs under primary care are from breeds with brachycephaly [5], this high level of breeds with brachycephaly in the comparator group may have effectively underestimated the odds ratios reported here for many predispositions of Pugs that are strongly associated with their brachycephalic conformation. The study selected a single year as the underlying time period for analysis in order to provide fixed temporal boundaries that could be replicated in other studies with other breeds and species to facilitate comparative studies. The year of 2016 was selected for study because the available study population approached 1 million dogs for that period. Reporting of mortality and of comparative disorder profiles for Pugs across multiple years were beyond the scope of the current study but are the focus of future planned VetCompass work.

## Conclusion

The current study highlights a series of common disorders that show either predisposition or protection in Pugs and that add to the evidence base urgently needed to reform this breed. Predispositions were shown to greatly outnumber protections, suggesting that there are some critical health and welfare challenges to overcome for Pugs. The widely differing health profiles between Pugs and other dogs in the UK suggest that the Pug has diverged to such an extent from mainstream dog breeds that the Pug breed can no longer be considered as a typical dog from the perspective of its disorder profile.

## Data Availability

The datasets generated during and/or analysed during the current study will be made available at the RVC Research Online repository.

## Abbreviations

BOAS: Brachycephalic obstructive airway syndrome
CI: Confidence interval
CFR: Craniofacial ratios
EPR: Electronic patient record
IQR: Interquartile range
KC: The Kennel Club
OR: Odds ratio
